# Effects of the *Epichloë* fungal endophyte symbiosis with *Schedonorus pratensis* on host grass invasiveness

**DOI:** 10.1002/ece3.1536

**Published:** 2015-06-04

**Authors:** Kruti Shukla, Heather A Hager, Kathryn A Yurkonis, Jonathan A Newman

**Affiliations:** 1 School of Environmental Sciences, University of GuelphGuelph, Ontario, Canada, N1G 2W1; 2 Department of Biology, University of North DakotaGrand Forks, North Dakota, USA, 58202

**Keywords:** *Festuca arundinacea*, *Festuca pratensis*, fungal endophyte, invasion, invertebrate community, *Lolium pratense*, meadow fescue, *Neotyphodium coenophialum*, *Neotyphodium uncinatum*, old-field, *Schedonorus phoenix*, tall fescue

## Abstract

Initial studies of grass–endophyte mutualisms using *Schedonorus arundinaceus* cultivar Kentucky-31 infected with the vertically transmitted endophyte *Epichloë coenophiala* found strong, positive endophyte effects on host-grass invasion success. However, more recent work using different cultivars of *S. arundinaceus* has cast doubt on the ubiquity of this effect, at least as it pertains to *S. arundinaceus–E. coenophiala*. We investigated the generality of previous work on vertically transmitted *Epichloë*-associated grass invasiveness by studying a pair of very closely related species: *S. pratensis* and *E. uncinata*. Seven cultivars of *S. pratensis* and two cultivars of *S. arundinaceus* that were developed with high- or low-endophyte infection rate were broadcast seeded into 2 × 2-m plots in a tilled, old-field grassland community in a completely randomized block design. *Schedonorus* abundance, endophyte infection rate, and co-occurring vegetation were sampled 3, 4, 5, and 6 years after establishment, and the aboveground invertebrate community was sampled in *S. pratensis* plots 3 and 4 years after establishment. Endophyte infection did not enable the host grass to achieve high abundance in the plant community. Contrary to expectations, high-endophyte *S. pratensis* increased plant richness relative to low-endophyte cultivars. However, as expected, high-endophyte *S. pratensis* marginally decreased invertebrate taxon richness. Endophyte effects on vegetation and invertebrate community composition were inconsistent among cultivars and were weaker than temporal effects. The effect of the grass–*Epichloë* symbiosis on diversity is not generalizable, but rather specific to species, cultivar, infection, and potentially site. Examining grass–endophyte systems using multiple cultivars and species replicated among sites will be important to determine the range of conditions in which endophyte associations benefit host grass performance and have subsequent effects on co-occurring biotic communities.

## Introduction

A number of species of European origin in the grass subfamily Poöideae have been developed and introduced for use as forage and amenity grasses throughout the temperate world. Many of these species can escape managed landscapes and compete with native species, reducing local biodiversity and altering ecosystem functioning (Rudgers et al. 2006; Rudgers and Clay 2007). Much of the success of these grasses, both within and outside of cultivation, is attributed to their evolutionary history with systemic, vertically transmitted, symbiotic fungal endophytes within the genus *Epichloë* (formerly *Neotyphodium;* Afkhami & Rudgers 2008; Schardl 2010; Leuchtmann et al. 2014).

These *Epichloë* species produce several classes of alkaloids that kill or deter insects and nematodes and, in some cases, are also toxic to vertebrate herbivores (e.g., Marks et al. 1991). Even in the absence of herbivory (e.g., in greenhouse experiments), *Epichloë*-infected hosts have exhibited greater vegetative growth and seed production (Schardl et al. 2004), greater nutrient uptake (e.g., Malinowski and Belesky 1999), greater tolerance of stressful conditions such as drought (e.g., Kannadan and Rudgers 2008), and greater competitive ability (e.g., Malinowski et al. 1997) than their uninfected counterparts. However, such benefits have not been universally demonstrated (Faeth et al. 2004), and some examples demonstrate at least a short-term cost to the plant host in some contexts (Richmond et al. 2003).

Because of the advantages that endophyte-infected individuals seem to have relative to uninfected conspecifics, one research question that has garnered significant interest is whether endophyte infection enhances the invasiveness of the host in a community context. Many glasshouse experiments have examined whether endophyte infection increases host competitive ability, and some have specifically examined broader plant community effects (e.g., Rudgers et al. 2005; Yurkonis et al. 2014a). Field experiments have been rarer.

Early field-based studies demonstrated strong effects of endophyte infection on host invasion. In large postagricultural plots seeded with *E. coenophiala*-infected (E+) or *E. coenophiala*-uninfected (E−) tall fescue *Schedonorus arundinaceus* (cultivar KY-31), all plots were dominated by the host grass after 4 years, but more so for E+ than E− plots (∼89% vs. 56%), and E+ plots had lower species richness (Clay and Holah 1999). In a nearby lowland site, invasion success was even greater (>97% for E+ and E−), and E+ plots had lower tree seedling abundance and richness than E− plots, attributed to higher vole (*Microtus* spp.) herbivory due to avoidance of E+ grass (Rudgers et al. 2007), as well as reduced arthropod abundance and diversity (Rudgers and Clay 2008) and altered arachnid community composition (Finkes et al. 2006).

However, several recent field studies, all using the *S. arundinaceus–E. coenophiala* system, have suggested a more context-dependent role for endophytes in invasion. At the same lowland site mentioned above, the effect of endophyte infection on host abundance depended on the cultivar 4–6 years after seeding for two different cultivars (Georgia-5, Jesup) that were E− or infected with one of two *E. coenophiala* strains, although host grass abundance was never >55% (Rudgers et al. 2010). The endophyte strain × cultivar combination also affected the relative abundance of forbs, and year had a strong and interactive effect on plant community measures (Rudgers et al. 2010). In small plots experimentally seeded with the same cultivars and endophyte strains, Yurkonis et al. (2014b) found that host grass mean abundance was <40%, and the effect of endophyte strain on plant and invertebrate community composition was year and cultivar dependent 4–5 years after seeding. In *S. arundinaceus* old-field plots (cultivar unknown), Spyreas et al. (2001) found a positive relation between endophyte infection frequency and species richness in mowed plots, but a negative relation in unmowed plots. Finally, in small plots experimentally seeded with 10 different E+ and E− forage and turf cultivars, Yurkonis et al. (2012) found that mean host grass abundance was again <40%, the plant community was more diverse in E+ than E− plots due to increased evenness resulting from the suppression of other dominant grass species, and the endophyte effect differed between forage and turf cultivars.

Given the results of these latter field studies and suggestions that community effects of grass–endophyte interactions are context dependent (e.g., Saikkonen et al. 2004, 2006), it is likely that host plant invasion success and subsequent effects will differ among species and cultivar–endophyte combinations. Here, we expand upon the *S. arundinaceus–E. coenophiala* invasion work by studying a pair of very closely related species, meadow fescue *S. pratensis* (Torrecilla and Catalán 2002) and its associated endophyte species *E. uncinata* (Schardl 2010; Leuchtmann et al. 2014). Most work on *S. pratensis–E. uncinata* has investigated agronomic conditions for its growth in managed pasture (Kölliker et al. 1999; Saari et al. 2009), but not its behavior in natural communities. In a forage context, *S. pratensis* cultivars are recognized as excellent competitors (Niemeläinen et al. 2001) and can increase in abundance over time when seeded into pastures (Saari et al. 2009). In natural populations, *S. pratensis* has moderate (∼40%) endophyte infection rates compared to *S. arundinaceus* (∼98%; Saikkonen et al. 2000). E+ and E− *S. pratensis* forage cultivars have been developed for use in northern latitudes, but it is unclear what community-scale effects these cultivars might have in natural systems. Therefore, we investigated the effects of seeding seven cultivars of *S. pratensis* differing in endophyte infection rates on the co-occurring plant and arthropod communities in an old-field ecosystem. We examined the hypothesis that fungal endophytes promote host grass abundance and reduce plant and arthropod diversity and composition, and compared responses in these communities to those sown with *S. arundinaceus* (KY-31 and Tulsa II) cultivars and unseeded control plots.

## Methods

### Study site

The experiment was established in an old-field community at the University of Guelph Turfgrass Institute and Environmental Research Center (Guelph, ON, Canada; 43°32′56″N, 80°12′39″W). Soil at the site is mainly a Guelph sandy loam (Brunisolic Gray-Brown Luvisol) developed on loam till. The plant community was previously dominated by the grasses *Poa pratensis* L. and *Elymus repens* L. Gould, and forbs such as *Taraxacum officinale* F.H. Wigg and *Cirsium arvense* L. Scop. Additional site details are provided by Yurkonis et al. (2012). Following Clay and Holah (1999), the site was prepared in early 2008 by tilling the ground twice to break the sod.

Daily mean temperature and total daily precipitation data for the study period were obtained from the Elora weather station (∼22 km northwest of the field site; 43°39′00″N, 80°25′00″W; Environment Canada; http://climate.weather.gc.ca). Temperature and precipitation were classified into overwinter (October–March) and growing season (April–September).

### Experimental design

One hundred 2 × 2 m plots separated by 0.5-m mowed aisles were established in an 826 m^2^ area in a completely randomized block design with 10 blocks. In each block, seven plots were seeded with one of seven *S. pratensis* cultivars, two plots were seeded with one of two *S. arundinaceus* cultivars, and one plot was left as an unseeded control. *Schedonorus* seed was hand-broadcast at a rate of 5 g/m^2^ in mid-June. Plots were covered with straw cloth to prevent local propagule migration and watered as needed until plants established from the added seed and the local propagule pool in early July. Thereafter, straw cloth was removed, vegetation within plots was left unmanaged, and interplot aisles were mown occasionally. No fertilizer or pesticides were applied.

Four *S. pratensis* E− cultivars (Antti, Fure, Ilmari, Kalevi) and three E+ cultivars (Inkeri, Kasper, Salten: S. Saari, University of Turku, Finland) widely used as forage grasses in Finland were selected for study. The *S. arundinaceus* cultivars included one E+ (KY-31: T. Philips, University of Kentucky, Lexington, KY) and one E− (Tulsa II: Seed Research of Oregon, Corvallis, OR) that are used extensively as forage in North America. Seeds were stored at −16°C upon arrival to minimize endophyte loss.

### Endophyte infection rate

To assess endophyte infection rate, we sampled the established *S. arundinaceus* in each plot in August 2010 and 2013, and *S. pratensis* in August 2010, 2011, and 2013. Nonreproductive tillers spaced at least 15 cm apart were collected (*N* = 1–11; limited by presence of tillers meeting the collection criterion) and stored in plastic bags at −8°C until they were tested for endophyte presence using an immunoblot assay (Phytoscreen field tiller endophyte detection kit; Agrinostics, Watkinsville, GA).

### Vegetation community

The vegetation was surveyed in July–August in each of 2010–2013 using nondestructive point-intercept sampling (Jonasson 1988; Brathen and Hagberg 2004; Yurkonis et al. 2012). Plots were sampled by placing eight narrow, steel pins vertically through the vegetation at 10-cm intervals along the plot diagonal. The first and last pins were located 0.5 m into the plot to avoid edge effects. We recorded the number of times each plant species touched each pin and used these data to calculate *Schedonorus* proportional abundance and three measures of plant biodiversity: richness (*S*), diversity (Simpson’s index 1/D), and evenness (1/D × 1/S). Plant biodiversity measures included *Schedonorus*. *Solidago* species of the *canadensis* complex (*S. altissima, S. canadensis,* and *S. gigantea*) could not be distinguished and were combined as *Solidago*.

### Invertebrate community

**Figure 1:**
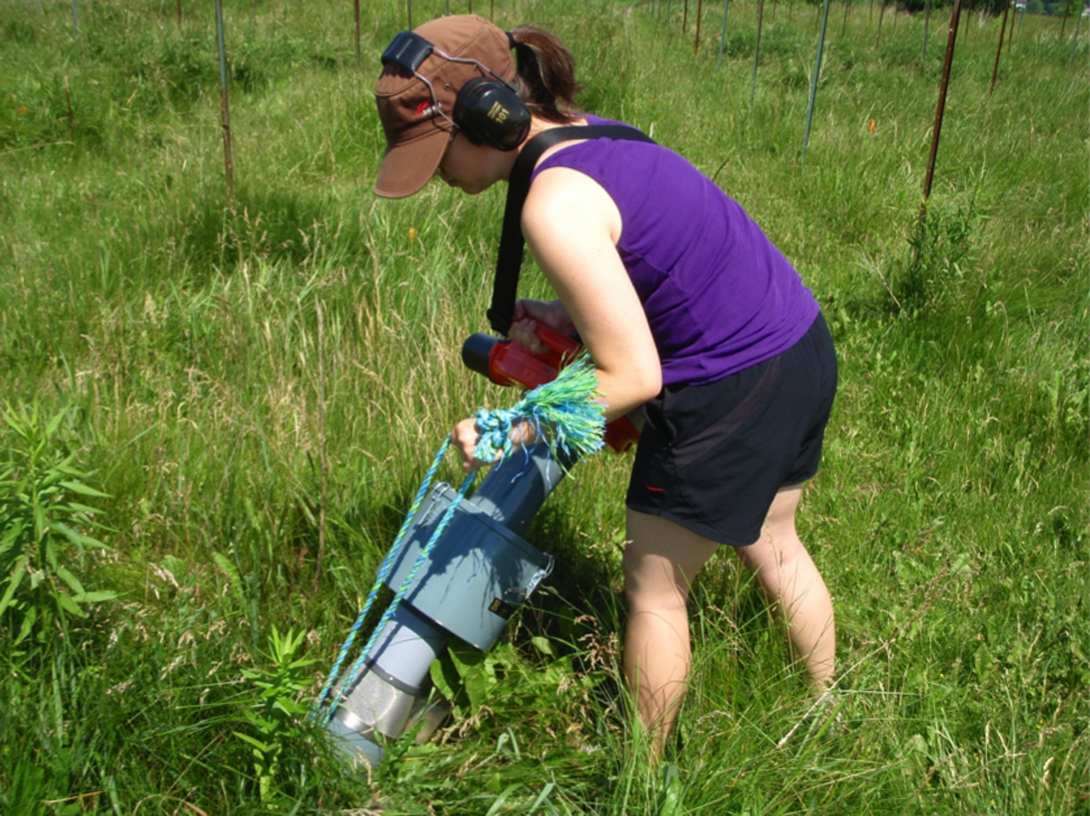
The invertebrate community associated with *Schedonorus pratensis, S. arundinaceus,* and unseeded plots was collected via vacuum sampling (shown above) in June 2010 and 2011. Photo credit: K. Shukla

Invertebrates were collected via vacuum sampling (Vortis Insect Suction Sampler; Burkard Manufacturing, Hertfordshire, UK) in all *S. pratensis* and unseeded plots in June 2010 and July 2011 (Fig.1). Samples were collected over a 60-s interval between 10 am and 2 pm from within the 1 × 1-m center of each plot (Helden and Leather 2004; Borges et al. 2009). Samples were stored at −23°C until individual invertebrates were counted and identified to family or, in some cases, order. These data were used to calculate plot invertebrate taxon richness, diversity, and evenness (as described for vegetation).

### Statistical analysis

All data are archived at the University of Guelph Agri-Environmental Research Data Repository (Shukla et al. 2015). The experiment was analyzed as a repeated-measures design using a split-plot analysis with cultivar as the whole plot factor and year as the subplot factor. Blocks were treated as a random effect; cultivar, year, and their interaction were fixed effects. All univariate and multivariate responses except climate were analyzed using this design.

#### Endophyte infection rate and *Schedonorus* abundance

To confirm cultivar status as E+ and E−, we tested for differences in *E. uncinata* endophyte infection rate in established *Schedonorus* (i.e., excluding control plots). Because *S. arundinaceus* infection rate was sampled in a subset of years in which *S. pratensis* infection rate was sampled, endophyte frequency was compared among all cultivars for 2010 and 2013, and then among *S. pratensis* cultivars for 2010, 2011, and 2013. We also examined *Schedonorus* proportional abundance in 2010–2013. We used generalized linear mixed models (SAS 9.4; SAS Institute, Cary, NC) to test the effects of cultivar, year, and their interaction on both response variables using Laplace maximum likelihood estimation. Over-dispersion was evaluated using the Pearson chi-square statistic divided by the degrees of freedom. Proportion of infected tillers and proportional abundance were tested using a binomial distribution and logit link function.

#### Biodiversity measures

We tested for effects of cultivar, year, and their interaction on community richness, diversity, and evenness using ANOVA with restricted maximum likelihood estimation followed by post hoc Tukey tests of significant effects. We used preplanned nonorthogonal contrasts to compare differences between species, and between endophyte status within *S. pratensis* (JMP 10; SAS Institute). Vegetation and invertebrate communities were analyzed separately. All individual invertebrate taxa were included in analyses. To satisfy ANOVA assumptions, response variables were Box-Cox transformed. We tested for correlation between vegetation and invertebrate richness, diversity, and evenness for *S. pratensis* E+, E−, and unseeded plots separately using Pearson correlation (r mass package; Venables and Ripley 2002; R Core Team 2014).

#### Community composition

We tested the effects of cultivar, year, and their interaction on the vegetation community (all cultivars plus unseeded; *S. pratensis* cultivars plus unseeded) and the invertebrate community using ordination. We used partial redundancy analysis (Canoco 5; Microcomputer Power, Ithaca, NY) to test cultivar and year effects on species abundances, using hierarchical permutations to maintain the split-plot form of the repeated-measures design (ter Braak and Smilauer 2012). Significance levels were determined using Monte Carlo estimation with 999 permutations.

For vegetation, rare species (<4 occurrences across 4 years) were omitted to avoid their undue influence on the analysis. *Schedonorus* was excluded as a response variable because it is confounded with cultivar treatment, but was included as a supplemental explanatory variable for visualization in biplots. For invertebrates, taxa that were present in both sample years and for which >80 individuals were detected were included, and taxa that had <80 individuals were combined into groups corresponding to their taxonomic order (McGarigal et al. 2000). For example, Musicidae, Camillidae, and Sciomyzidae had fewer than 80 individuals and were combined to create the group “Other Diptera”. Vegetation and invertebrate data were log(*X* + 1)-transformed and centered for analysis.

#### Climate

Daily precipitation was converted to total weekly precipitation to reduce the number of zeros and was Box-Cox transformed to meet statistical assumptions. We used two-factor ANOVA (JMP 10) to test the effects of year, season, and their interaction on daily mean temperature and total weekly precipitation, followed by post hoc Tukey tests of significant effects.

## Results

### Endophyte infection rate and *Schedonorus* abundance

**Figure 2:**
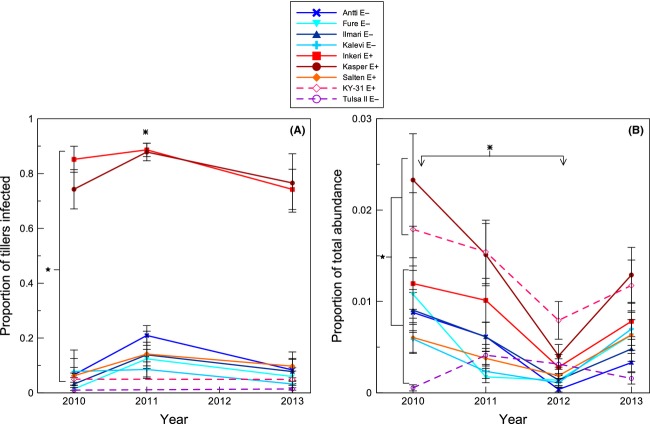
Proportion of *Schedonorus pratensis* (solid lines) and *S. arundinaceus* (broken lines) tillers that tested positive for endophyte presence (A) and *Schedonorus* relative abundance over time (B) in an old-field community. Means and standard errors are back transformed from generalized linear mixed models using inverse link functions and the delta method. *S. arundinaceus* cultivars were not tested for endophyte presence in 2011; no cultivars were tested in 2012. Stars and asterisks indicate significant differences between cultivars and years, respectively (post hoc Tukey tests, *P* < 0.05).

The proportion of infected tillers differed significantly among cultivars (*S. pratensis F*_6,63_ = 38.46, *P* < 0.0001; *S. arundinaceus* and *S. pratensis F*_8,81_ = 19.06, *P* < 0.0001), years (*S. pratensis F*_2,125_ = 14.69, *P* < 0.0001), and with their interaction (*S. pratensis F*_12,125_ = 1.99, *P* = 0.03; *S. arundinaceus* and *S. pratensis F*_8,80_ = 2.5, *P* = 0.02). The infection rate was significantly greater for the E+ cultivars Inkeri and Kasper than for all other cultivars (Fig.2A). Both Salten and KY-31, which we obtained as E+ cultivars, had very low infection rates in the field that did not differ from those of E− cultivars. Therefore, we treat these as E− cultivars in subsequent analyses. Infection rate for *S. pratensis* was greater in 2011 than in 2010 and 2013 (Fig.2A).

*Schedonorus* spp. abundance differed significantly among cultivars (*F*_8,72_ = 10.83, *P* < 0.0001) and, as with endophyte frequency, the magnitude of these differences varied among years (Year: *F*_3,243_ = 29.33, *P* < 0.0001; Cultivar × Year: *F*_24,243_ = 4.44, *P* < 0.0001). Kasper and KY-31 were similar to Inkeri and were consistently more abundant than the remaining cultivars (Fig.2B). Temporally, *Schedonorus* spp. were most abundant in 2010 and composed the smallest proportion of the vegetation in 2012 (Fig.2B). The *S. arundinaceus* cultivar Tulsa II most notably varied from the others in its temporal trend, occurring at its highest abundance in 2011.

### Community diversity

Including *Schedonorus,* 24 plant species were encountered during point-intercept sampling. Vegetation richness, diversity, and evenness differed significantly among years (Table1). Richness was greatest in 2011, followed by 2010 and 2013 and then 2012 (Tukey, *P* < 0.02; see Fig. S1 in Supporting Information). Diversity was greater in 2010 and 2011 than in 2012 and 2013 (*P* < 0.0001; Fig. S1). In contrast, evenness was greater (more even) in 2010, followed by 2012 and then 2011 (*P* < 0.035); evenness in 2013 did not differ from that in 2011 or 2012 (Fig. S1). Cultivar significantly affected vegetation evenness and marginally affected richness (Table1). *S. arundinaceus* reduced plot evenness relative to *S. pratensis* and unseeded plots, and Kasper plots were more even than either of the *S. arundinaceus* plots (Tukey, *P* < 0.003; Fig. S1). Both *Schedonorus* species increased local species richness relative to unseeded plots, and this effect was stronger in E+ than in E− *S. pratensis* plots (Table1; Fig. S1).

**Table 1:** Univariate ANOVA results for effects of *Schedonorus pratensis* and *S. arundinaceus* on vegetation diversity measures. E+ and E− contrasts are for *S. pratensis* only. Response variables were Box-Cox transformed for analysis. Bold font indicates significant effects or contrasts

Source	df	Richness		Diversity		Evenness	
		*F*	*P*	*F*	*P*	*F*	*P*
Cultivar	9,81	1.82	0.078	1.60	0.13	3.21	**0.002**
*S.arun* vs. *S.prat*	1,81	0.03	0.85	0.19	0.66	19.97	**<0.0001**
*S.arun* vs. unseeded	1,81	4.06	**0.05**	0.01	0.91	3.92	**0.05**
*S.prat* vs. unseeded	1,81	4.68	**0.03**	0.22	0.64	1.17	0.28
Within *S.prat*							
E+ vs. E−	1,81	2.65	0.11	1.71	0.19	2.29	0.13
E+ vs. unseeded	1,81	7.20	**0.009**	1.09	0.30	2.84	0.10
E− vs. unseeded	1,81	3.08	0.08	0.03	0.87	0.53	0.47
Year	3,81	21.83	**<0.0001**	27.46	**<0.0001**	13.95	**<0.0001**
Cultivar × Year	27,270	1.19	0.24	1.24	0.20	1.35	0.12

A total of 82,054 invertebrates were identified (33,494 in 2010; 48,560 in 2011), comprising 57 families in 10 orders. The effect of *S. pratensis* cultivars on invertebrate richness, diversity, and evenness was nonsignificant (Table2). However, E+ plots were marginally less taxonomically rich (∼1 taxon) and more even than E− plots (*P* = 0.08 and 0.11, respectively; Table2, Fig. S2). Neither E+ nor E− plots differed from unseeded plots in richness or evenness. Invertebrate diversity and evenness varied between years, with more diverse taxa more evenly distributed in 2011 than in 2010 (Table2, Fig. S2). There was no cultivar × year interaction (Table2).

**Table 2:** Univariate ANOVA results for effects of *Schedonorus pratensis* on invertebrate diversity measures. Response variables were Box-Cox transformed for analysis. Bold font indicates significant effects or contrasts

Source	df	Richness		Diversity		Evenness	
		*F*	*P*	*F*	*P*	*F*	*P*
Cultivar	7,63	1.18	0.33	0.86	0.54	1.12	0.36
E+ vs. E−	1,63	3.28	0.08	0.23	0.63	2.69	0.11
E+ vs. unseeded	1,63	0.22	0.64	1.34	0.25	1.33	0.25
E− vs. unseeded	1,63	0.73	0.40	0.86	0.36	0.002	0.97
Year	1,72	0.42	0.52	48.07	**<0.0001**	24.11	**<0.0001**
Cultivar × Year	1,72	0.78	0.61	0.44	0.87	0.60	0.76

There was no correlation between vegetation and invertebrate richness, diversity, or evenness in *S. pratensis* E+ (*R* = 0.03, *P* = 0.87; *R* = 0.09, *P* = 0.59; *R* = −0.14, *P* = 0.39, respectively), E− (*R* = −0.04, *P* = 0.66; *R* = 0.01, *P* = 0.89; *R* = −0.13, *P* = 0.17, respectively), or unseeded plots (*R* = −0.11, *P* = 0.64; *R* = −0.05, *P* = 0.83; *R* = 0.0002, *P* = 1.0, respectively).

### Community composition

**Figure 3:**
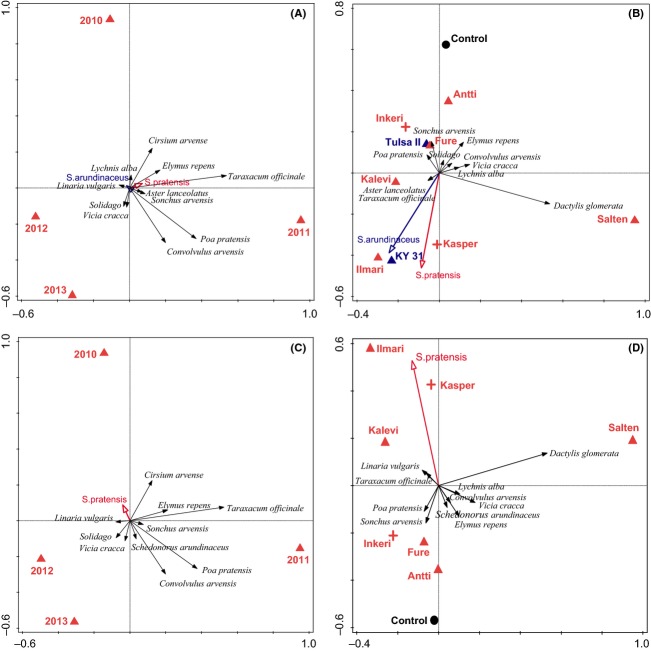
Biplots based on partial redundancy analyses of the vegetation composition with respect to the effect of year (A,C) and *Schedonorus* cultivar (B,D) treatment. (A,B) All cultivars, the 10 best fitting of 13 plant species are displayed. (C,D) *S. pratensis* cultivars, the 10 best fitting of 12 plant species are displayed. Blue = *S. arundinaceus* cultivars, red = *S. pratensis* cultivars, triangle = low-endophyte frequency (E−), + = high-endophyte frequency (E+).

The vegetation and invertebrate community composition differed among years, and cultivar marginally affected vegetation composition within the reduced *S. pratensis* data set (Table3). In the analysis including *S. arundinaceus*, year explained nearly twice the variation in vegetation composition as cultivar (10.5% vs. 5.5%; Table3). In the year biplot, axis 1 separated 2011 and axis 2 separated 2010 from the other years (Fig.3A). *Taraxacum officinale* and *Cirsium arvense* were more abundant in 2010 and 2011, and *Poa pratensis* in 2011, than in 2012 and 2013. *Convolvulus arvensis* was less abundant in 2010 than in the other years (see Table S1 for species loadings). The supplementary explanatory variables, *S. arundinaceus* and *S. pratensis* abundance, were not correlated with either of the first two ordination axes.

**Table 3:** ANOVA results from partial redundancy analyses of vegetation composition for all cultivars (*S. pratensis, Schedonorus arundinaceus,* and unseeded plots) and of vegetation and invertebrate community abundances for *S. pratensis* cultivars (*S. pratensis* and unseeded plots). Bold font indicates significant effects

Community	Source	df	Total SS	Pseudo-*F*	*P*	% variation^1^
All cultivars						
Vegetation	Block	9	0.102931			
	Cultivar	9	0.0490333	2.4	0.13	5.5
	Year	3	0.0892696	14.8	**0.001**	10.5
	C × Y	27	0.0331225	0.6	0.855	4.4
	Residual		0.725644			
*S. pratensis* cultivars						
Vegetation	Block	9	0.106186			
	Cultivar	7	0.0516785	2.7	0.075	5.8
	Year	3	0.090536	12.0	**0.001**	10.8
	C × Y	21	0.0324174	0.6	0.851	3.8
	Residual		0.719182			
Invertebrate	Block	9	0.128059			
	Cultivar	7	0.0293377	0.7	0.766	3.4
	Year	1	0.179098	38.3	**0.001**	21.3
	C × Y	7	0.0323015	1.0	0.448	4.9
	Residual		0.631204			

1 Percent variation in community abundance data explained by the model term.

In the cultivar biplot, axis 1 separated Salten (E−) from the remaining cultivars, and axis 2 separated unseeded plots from Kasper (E+), Ilmari (E−), and KY-31 (E−) plots (Fig.3B; see Fig. S3 for standard errors of plot scores). There was no clear separation of cultivars with respect to *Schedonorus* species or endophyte status. *S. pratensis* abundance was highly associated with Kasper and Ilmari plots, and *S. arundinaceus* was highly associated with KY-31 plots. *S. pratensis* and *S. arundinaceus* were negatively associated with unseeded and Antti plots, and with the dominant grasses *Poa pratensis* and *Elymus repens* and the thistle *Sonchus arvensis. Dactylis glomerata* was highly associated with Salten (Fig.3B).

In the analysis excluding *S. arundinaceus*, year again explained nearly twice as much variation in vegetation composition as cultivar (10.8% vs. 5.8%; Table3). As in the full analysis, axis 1 of the year biplot separated 2011, axis 2 separated 2010 from the other years, and vegetation associations were similar (Fig.3C; see Table S2 for species loadings). *S. pratensis* was weakly correlated with axis 2. In the cultivar biplot, axis 1 separated Salten from the remaining cultivars, axis 2 separated control plots from Kasper and Ilmari plots, and *Dactylis glomerata* was highly associated with Salten (Fig.3D; see Fig. S3 for standard errors of plot scores). *S. pratensis* abundance was positively associated with Kasper and Ilmari plots and negatively associated with control, Antti, and Fure plots, and with *Poa pratensis, Elymus repens,* and *Sonchus arvensis* (Fig.3D; Table S2). There was no clear separation of cultivars with respect to endophyte status.

**Figure 4:**
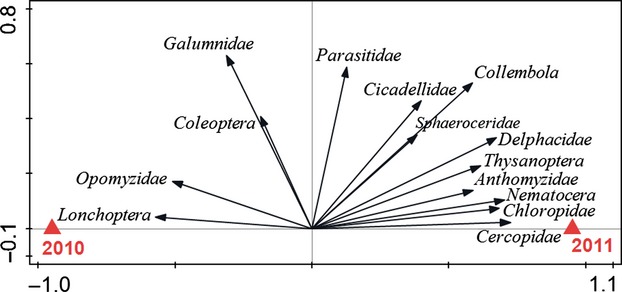
Biplots based on partial redundancy analyses of invertebrate community abundance in an old field with respect to the effect of year. The 14 best fitting of 27 invertebrate groups are displayed.

For the invertebrate community, year explained 21.3% of the variation in taxon abundances and there was no effect of *S. pratensis* cultivar (Fig.4). Invertebrates were generally more abundant in 2011 than in 2010 (Table S3).

### Climate

Weekly total precipitation was greater in 2013 than in 2012 (*F*_3,202_ = 2.84, *P* = 0.04), but did not differ between seasons (Season: *F*_1,202_ = 1.37, *P* = 0.2; Year × Season: *F*_3,202_ = 1.08, *P* = 0.4). The growing season was warmer than the overwintering season (*F*_1,1435_ = 2488.3, *P* < 0.0001), and temperature differed among years (*F*_3,1435_ = 7.54, *P* < 0.0001) and with their interaction (*F*_3,1435_ = 5.23, *P* = 0.0014). Overall, 2011 and 2013 were colder than 2012, and this was mainly due to differences in overwinter temperatures (Fig. S4).

## Discussion

Association with vertically transmitted *Epichloë* endophytes has generally been thought to benefit host grass fitness and provide host grasses with a competitive advantage over other plant species. However, we found that endophyte infection did not enable *S. pratensis* cultivars to achieve high abundance in an old-field community. Three to six years after seeding, neither *Schedonorus* species became dominant in the plant community, regardless of endophyte status, comprising ≤6.3% of the vegetation (on average, 1.1% in E+ plots [*S. pratensis*] and 0.55% in E− plots [*S. pratensis* and *S. arundinaceus*]). Whether low abundance was due to low establishment or low survival is unknown. It is notable, however, that one E+ *S. pratensis* cultivar (Kasper) and one E− *S. arundinaceus* cultivar (KY-31) were more abundant than all other cultivars (one E+ *S. pratensis*, five E− *S. pratensis*, and one E− *S. arundinaceus*), indicating that abundance was not related to species or endophyte effects alone.

Despite the low *Schedonorus* abundance in our plots, comparable work with five different *S. arundinaceus* forage cultivars at our site found that mean abundance ranged from >20% to 40% 4 years after seeding (Yurkonis et al. 2012), regardless of endophyte status. In contrast, although sampling methods differed, studies of *S. arundinaceus* KY-31 in Indiana, USA, found that even low-endophyte grass mean abundance was >56% and up to 97% of total plant biomass (Rudgers et al. 2007). Thus, site-specific effects may be stronger than species, cultivar, and endophyte effects, suggesting that future work should be replicated in a variety of locations. Site-specific effects could be related to differences in climate, soil moisture and nutrient availability, year, or other factors. For example, whereas we obtained seeds from Finland and the United States, the seeds used in some previous experiments came from plants that had been grown in those areas for a number of years (Clay and Holah 1999; Rudgers et al. 2007). Thus, the plants were not necessarily locally adapted to the environmental conditions, soil characteristics, or vegetation community at our experimental location, which could have affected their performance.

### Effects on community diversity

Even though *S. pratensis* abundance was inconsistent with respect to endophyte status, there was an endophyte effect on community diversity, although the response was small for both plants and invertebrates. For plant communities, the effect was in the opposite direction from that expected: species richness was greater in E+ than unseeded plots and marginally significantly greater for E− versus unseeded plots and E+ versus E− plots. Communities that received *S. pratensis* or *S. arundinaceus* seed had ∼0.4 species more than those that did not. These results suggest that the *Schedonorus* species became incorporated in the plant community, rather than displacing other species and becoming dominant as expected, and may have done so by reducing the abundance of other dominant species. Our analyses of community composition indicate that *Schedonorus* spp. abundance was negatively correlated with that of the other dominant grasses in the ecosystem (*Poa pratensis* or *Elymus repens;* Fig.3B and D). There is corroborating evidence that this was also the case for five forage and five turf *S. arundinaceus* cultivars at the same site (Yurkonis et al. 2012). The success of *Schedonorus* invasion may depend on interactions with the particular competing plant community (Saikkonen et al. 2000; Takai et al. 2010; Yurkonis et al. 2012, 2014a,b). Given that some previous *S. arundinaceus* studies have been performed in communities that were originally dominated by *S. arundinaceus* (e.g., Clay and Holah 1999; Spyreas et al. 2001), legacy effects might explain its greater abundance at those sites than at our site, regardless of endophyte status.

We are the first to quantify changes in the invertebrate community as a result of effects on the vegetation community for *S. pratensis*–*E. uncinata*. We expected that invertebrate richness would be lower in E+ plots because *E. uncinata* produces loline alkaloids, which are thought to deter invertebrates (Bush et al. 1997; Schardl et al. 2004). Even though *S. pratensis* abundance was low such that herbivores would likely encounter these individuals with low frequency, we did detect marginally lower invertebrate taxon richness in E+ than E− plots. In addition, plant and invertebrate diversity measures were uncorrelated within plot types, suggesting that invertebrate diversity is affected by factors other than plant community structure in these plots. It is difficult to place our invertebrate community results in the context of previous work. Whereas our host grass comprised only a small proportion of the vegetation community, studies with other grasses were based on invertebrate communities sampled from vertically transmitted *Epichloë*-associated grass species grown individually (Faeth and Shochat 2010; Jani et al. 2010; Vesterlund et al. 2011) or when they comprised the majority of the vegetation (Lemons et al. 2005; Finkes et al. 2006; Rudgers and Clay 2008). Nevertheless, previous results have ranged from higher invertebrate diversity on two E+ native grasses in southwestern USA (Faeth and Shochat 2010; Jani et al. 2010) to little effect of endophyte status for *S. arundinaceus* from wild European populations and U.S. cultivar KY-31 grown in Europe (Vesterlund et al. 2011) and to strong negative effects for E+ *S. arundinaceus* KY-31 in Indiana, USA (Lemons et al. 2005; Finkes et al. 2006; Rudgers and Clay 2008). The latter strong effects were thought to be caused by a decrease in plant richness, and thus herbivore resources, due to the dominant E+ grass (Rudgers and Clay 2008). Clearly, work remains to determine the mechanisms underlying the different effects of various grass–endophyte associations on both their herbivore consumers and invertebrate communities in general, particularly when the grasses occur in mixed plant communities.

Previous work with *S. arundinaceus* KY-31 found that herbivory by voles (*Microtus* spp.) reduced plant diversity in E+ compared to E− plots because voles selectively consumed woody species and avoided the dominant E+ plants within their territories (Rudgers et al. 2007). Although our experimental plots could have been smaller than the resident meadow vole (*Microtus pennsylvanicus*) territories, allowing voles to select plants outside the plots, we expect that selective vole herbivory is not a major factor in our experiment for two reasons. First, endophyte-infected *S. pratensis* produces only loline alkaloids, which are not generally deterrent to mammalian herbivores (Bush et al. 1997; Schardl et al. 2004), and both *S. arundinaceus* cultivars had low-endophyte infection rates, making them also not deterrent to mammals. Second, if voles were selectively avoiding consumption of E+ *S. pratensis*, we would expect to detect a much stronger effect of endophyte presence on host abundance, regardless of vole territory size.

Although there were some differences in plant and invertebrate diversity and composition among cultivars and between high- and low-endophyte plots, our experimental design does not allow testing the cultivar × endophyte interaction. However, it does allow testing a larger variety of the E+ and E− cultivars that are often used in practical applications. In future, testing various E+ cultivars with the same cultivars that have had the endophyte removed will be important because community effects appear to be specific to some cultivar–endophyte combinations (e.g., Fig.3B and D). This often decreases the scope of inference, however, because the dramatic increase in the number of experimental plots required usually means that fewer cultivars can be compared.

### Effects on community composition

Similar to the results of Yurkonis et al. (2012, 2014a,b), effects of *Schedonorus* species on plant community composition depended more on the particular cultivar than on the species or endophyte status, as indicated by our multivariate analysis. Cultivars could not be grouped based on species or endophyte effects on plant community composition. In particular, Kasper (E+ *S. pratensis*), Ilmari (E− *S. pratensis*), and KY-31 (E− *S. arundinaceus*) plots had similar plant community composition, but differed with respect to species and endophyte status. Antti (E− *S. pratensis*) and Inkeri (E+ *S. pratensis*) were most similar to unseeded plots. It is notable that the two *S. arundinaceus* cultivars, although both low-endophyte, had very different effects on plant community composition. Given that KY-31 and Kasper had the highest abundances of all cultivars, it may be that host grass abundance has a stronger effect than endophyte status on plant community composition. However, it is unclear why Ilmari would have similar effects because this cultivar was not highly abundant. Nevertheless, cultivar differences did not translate to effects on invertebrate community composition.

Differences among cultivars may be related to the purpose for which each cultivar was bred, for example, forage, pasture, or amenity purposes (Hoveland 2009). Developing cultivars that exhibit specific characteristics or functionality might decrease the phenotypic plasticity of the plant and the range of conditions to which it could adapt (Kölliker et al. 1999; Ghalambor et al. 2007). Forage cultivars are selected to coexist in mixed plant communities and so should be successful in natural communities (Saikkonen et al. 1998; Kölliker et al. 1999; Saari and Faeth 2012). However, their invasive potential might be reduced when they are introduced to areas where specific requirements are not met. For example, a meta-analysis of *Epichloë*–grass symbioses indicates that high soil nutrient availability is linked to greater endophyte benefits (Saikkonen et al. 2006); in contrast, soil nutrient availability at our site is low (Yurkonis et al. 2012).

The strong temporal effect for all response variables could be related to climatic effects on plant growth and survival. Overall, plant richness and diversity and *Schedonorus* abundance were low in 2012, suggesting the occurrence of a large-scale event. This may have been related to the cold overwinter temperatures in 2011 or somewhat lower levels of precipitation in 2012. An unusually high vole population in 2012 might also have affected overall plant abundances (Hager and Stewart 2013). Further investigation is required to determine the mechanisms underlying strong temporal variation, and our results stress the importance of long-term experiments in examining host-endophyte effects.

Finally, it is unreasonable to assume that *S. pratensis* and *S. arundinaceus* are the only species within our community that are associated with microbial symbionts (Rudgers et al. 2009). Our communities were dominated mainly by other graminoids, but also forbs and legumes, some of which are associated with other types of endophyte (Saikkonen et al. 2010). Very little is currently known about the endophyte status of, and its effects in, other plant species. Future work examining the prevalence and competitive ability of other endophyte-associated species in comparison with our focal species could provide insight into potential species and habitat interactions.

## Conclusion

Initial studies of grass–endophyte mutualisms using *S. arundinaceus–E. coenophiala* cultivar KY-31 found strong, positive endophyte effects on the host grass invasion success. However, our evidence indicates that endophyte presence does not result in similar benefits to the host grass in other poöid–*Epichloë* symbiotic associations. The effect of the grass–*Epichloë* symbiosis is not generalizable, but rather species, cultivar, and endophyte specific. Effects of site conditions such as the composition of competing vegetation, resource availability, and climate will also make it difficult to forecast the outcome of a poöid–*Epichloë* invasion. Examining multiple grass–endophyte systems using multiple cultivars, species, and sites will be important to determine the range of conditions in which such endophyte associations benefit host grass performance and have subsequent effects on the co-occurring biotic communities.

## Supporting Information

**Figure S1.** Significant effects and contrasts for univariate tests of plant richness, diversity and evenness.

**Figure S2.** Significant effects and contrasts for univariate tests of invertebrate taxon richness, diversity and evenness.

**Figure S3.** Variation in plot scores for cultivar effect on plant community composition.

**Figure S4.** Significant effects for temperature and precipitation.

**Table S1.** Species loadings for year and cultivar effects from partial redundancy analysis of the plant community for the complete data set.

**Table S2.** Species loadings for year and cultivar effects from partial redundancy analysis of the plant community for the *Schedonorus pratensis* data set.

**Table S3.** Species loadings for year and cultivar effects from partial redundancy analysis of the invertebrate community for the *S. pratensis* data set.
