# Supplementary material for: Effects of the Epichloë fungal endophyte symbiosis with Schedonorus pratensis on host grass invasiveness
Source: Ecol Evol. 2015 Jun 4;5(13):2596–607. doi: 10.1002/ece3.1536 (PMC4523356; doi:10.1002/ece3.1536)
Supplement: Supplementary file 1 [file ece30005-2596-sd1.docx]

**Fig. S1.** Significant effects and contrasts for univariate tests of plant species richness (a-c), plant diversity (d), and plant evenness (e-g). Lowercase letters indicate treatment differences within the effects of Year and Cultivar (post-hoc Tukey tests, P < 0.05). Asterisks indicate significant pre-planned contrasts (P < 0.05).
