# Supplementary material for: Effects of the Epichloë fungal endophyte symbiosis with Schedonorus pratensis on host grass invasiveness
Source: Ecol Evol. 2015 Jun 4;5(13):2596–607. doi: 10.1002/ece3.1536 (PMC4523356; doi:10.1002/ece3.1536)
Supplement: Supplementary file 2 [file ece30005-2596-sd2.docx]

**Fig. S2.** Significant effects and contrasts for univariate tests of invertebrate taxon richness (a), invertebrate taxon diversity (b), and invertebrate taxon evenness (c,d). Lowercase letters indicate treatment differences within Year (post-hoc Tukey tests, P < 0.05). Crosses indicate marginally significant pre-planned contrasts (a, P = 0.08; d, P = 0.11).
