# Supplementary material for: Effects of the Epichloë fungal endophyte symbiosis with Schedonorus pratensis on host grass invasiveness
Source: Ecol Evol. 2015 Jun 4;5(13):2596–607. doi: 10.1002/ece3.1536 (PMC4523356; doi:10.1002/ece3.1536)
Supplement: Supplementary file 3 [file ece30005-2596-sd3.docx]

**Fig. S3.** Plot centroids and standard error of plot scores for cultivar effect on plant community composition. (A) Analysis with all *Schedonorus pratensis* and *S. arundinaceus* cultivars. (B) Analysis with *S. pratensis* cultivars. Plots are not scaled to response variables (as in Fig. 2).
