# Supplementary material for: Effects of the Epichloë fungal endophyte symbiosis with Schedonorus pratensis on host grass invasiveness
Source: Ecol Evol. 2015 Jun 4;5(13):2596–607. doi: 10.1002/ece3.1536 (PMC4523356; doi:10.1002/ece3.1536)
Supplement: Supplementary file 4 [file ece30005-2596-sd4.docx]

**Fig. S4.** Effects of Year x Season on mean daily temperature (a) and effects of Year on total weekly precipitation (b). Overwinter = October–March; Growing season = April–September. Lowercase letters indicate significant differences (post-hoc Tukey tests, P < 0.05).
