# Supplementary material for: Effects of the Epichloë fungal endophyte symbiosis with Schedonorus pratensis on host grass invasiveness
Source: Ecol Evol. 2015 Jun 4;5(13):2596–607. doi: 10.1002/ece3.1536 (PMC4523356; doi:10.1002/ece3.1536)
Supplement: Supplementary file 5 [file ece30005-2596-sd5.docx]

**Table S1.** Species loadings for year and cultivar effects from partial redundancy analysis of the plant community for the *Schedonorus arundinaceus* and *S. pratensis* combined data set.

|  | Year | | Cultivar | |
| --- | --- | --- | --- | --- |
| Species | Axis 1 | Axis 2 | Axis 1 | Axis 2 |
| *Arctium minus* | 0.0059 | 0.0354 | -0.043 | 0.0018 |
| *Aster lanceolatus* | 0.0694 | -0.0235 | -0.0556 | -0.0357 |
| *Cirsium arvense* | 0.1259 | 0.2209 | 0.0202 | -0.0021 |
| *Convolvulus arvensis* | 0.1994 | -0.3034 | 0.0617 | 0.0479 |
| *Dactylis glomerata* | 0.0323 | 0.003 | 0.5375 | -0.1512 |
| *Elymus repens* | 0.1682 | 0.0995 | 0.1174 | 0.1503 |
| *Linaria vulgaris* | -0.0591 | 0.0163 | -0.0576 | -0.0069 |
| *Lychnis alba* | 0.0073 | 0.0717 | 0.0972 | 0.0282 |
| *Poa pratensis* | 0.369 | -0.2824 | -0.0608 | 0.0876 |
| *Solidago canadensis* complex | -0.034 | -0.1027 | 0.0209 | 0.0628 |
| *Sonchus arvensis* | 0.0865 | -0.0326 | -0.039 | 0.1532 |
| *Taraxacum officinale* | 0.5373 | 0.069 | -0.0552 | -0.0384 |
| *Vicia cracca* | -0.0165 | -0.1064 | 0.148 | 0.0417 |
