# Supplementary material for: Effects of the Epichloë fungal endophyte symbiosis with Schedonorus pratensis on host grass invasiveness
Source: Ecol Evol. 2015 Jun 4;5(13):2596–607. doi: 10.1002/ece3.1536 (PMC4523356; doi:10.1002/ece3.1536)
Supplement: Supplementary file 6 [file ece30005-2596-sd6.docx]

**Table S2.** Species loadings for year and cultivar effects from partial redundancy analysis of the plant community for the *Schedonorus pratensis* data set.

|  | Year | | Cultivar | |
| --- | --- | --- | --- | --- |
| Species | Axis 1 | Axis 2 | Axis 1 | Axis 2 |
| *Cirsium arvense* | 0.1249 | 0.2244 | 0.0207 | -0.0149 |
| *Convolvulus arvensis* | 0.1988 | -0.298 | 0.0581 | -0.0719 |
| *Dactylis glomerata* | 0.0357 | 0.006 | 0.5316 | 0.1382 |
| *Elymus repens* | 0.2113 | 0.0598 | 0.1011 | -0.1299 |
| *Linaria vulgaris* | -0.0802 | -0.0074 | -0.08 | 0.0656 |
| *Lychnis alba* | -0.0017 | 0.024 | 0.1042 | -0.0364 |
| *Poa pratensis* | 0.375 | -0.2685 | -0.0705 | -0.1084 |
| *Schedonorus arundinacea* | 0.0361 | -0.1019 | 0.042 | -0.0929 |
| *Solidago* | -0.078 | -0.097 | 0.0252 | -0.0177 |
| *Sonchus arvensis* | 0.0749 | -0.021 | -0.0625 | -0.1583 |
| *Taraxacum officinale* | 0.5261 | 0.076 | -0.0602 | 0.0538 |
| *Vicia cracca* | -0.0268 | -0.1132 | 0.1793 | -0.0715 |
