# Supplementary material for: Effects of the Epichloë fungal endophyte symbiosis with Schedonorus pratensis on host grass invasiveness
Source: Ecol Evol. 2015 Jun 4;5(13):2596–607. doi: 10.1002/ece3.1536 (PMC4523356; doi:10.1002/ece3.1536)
Supplement: Supplementary file 7 [file ece30005-2596-sd7.docx]

**Table S3.** Species loadings for year and cultivar effects from partial redundancy analysis of the invertebrate community for the *Schedonorus pratensis* data set.

|  | Year | |
| --- | --- | --- |
| Invertebrate taxon group | Axis 1 | Axis 2 |
| Opomyzidae | -0.5098 | 0.1713 |
| Anthomyzidae | 0.5894 | 0.1376 |
| Chloropidiae | 0.685 | 0.0732 |
| Phoridae | 0.3551 | 0.2067 |
| Sphaeroceridae | 0.3845 | 0.3398 |
| Nematocera | 0.7046 | 0.1034 |
| Lonchopteridae | -0.5705 | 0.0425 |
| Drosophilidae | 0.419 | 0.0531 |
| Other Diptera | 0.4106 | 0.0859 |
| Aphididae | 0.0537 | 0.3584 |
| Pentomidae | -0.0858 | 0.0569 |
| Cicadellidae | 0.3996 | 0.4646 |
| Cercopidae | 0.7254 | 0.0221 |
| Nabidae | 0.0864 | 0.3002 |
| Delphacidae | 0.6752 | 0.3301 |
| Other Hemipterans | 0.1214 | 0.0416 |
| Chalcidoidea | 0.1624 | 0.2017 |
| Other Hymenoptera | 0.0206 | 0.1007 |
| Coleoptera | -0.1878 | 0.4075 |
| Snails (Gastropoda) | -0.3119 | 0.0586 |
| Lepidoptera + Orthoptera | -0.0252 | 0.1877 |
| Araneae + Opiliones | 0.228 | 0.3483 |
| Galumnidae | -0.3107 | 0.6301 |
| Parasitidae | 0.1282 | 0.5878 |
| Thysanoptera | 0.6155 | 0.228 |
| Collembola | 0.5868 | 0.5298 |
| Other | -0.2382 | 0.2784 |
